# Perceiving intimidation through kinematic cues in men’s gait

**DOI:** 10.1038/s41598-025-16400-y

**Published:** 2025-09-29

**Authors:** K. McCarty, C. Leslie, A. Anguera de la Rosa, T. K. Saxton, P. L. Cornelissen

**Affiliations:** 1 Department of Psychology, Northumbria University, Newcastle Upon Tyne, UK; 2 Department of Social Sciences, Northumbria University, Newcastle Upon Tyne, UK

**Keywords:** Physical dominance, Formidability, Biomechanics, Gait analysis, Social perception, Dynamic cues, Trait aggression, Human behaviour, Sexual selection

## Abstract

Male intrasexual competition has shaped the evolution of sexually dimorphic traits, influencing both physical and cognitive mechanisms for assessing physical dominance and formidability. While previous research has examined static visual cues such as height and upper body musculature to male formidability, the role of dynamic cues remains underexplored. We used 3D motion capture to investigate biomechanical markers that drive perceptions of male physical dominance. Fifty-two male participants’ walking gaits were recorded, and their upper-body strength, anthropometric measures, and self-reported aggression were assessed. A separate group of 137 raters evaluated the dominance of these walking patterns. Results revealed that one dynamic cue, lateral torso oscillation (sway) and one postural cue, shoulder abduction, were correlated with physical dominance as well as with anthropometric measures (e.g., height, chest size) and trait aggression scores. A linear mixed-effects model demonstrated that sway, shoulder abduction and body size/strength all contributed independently to predicting physical dominance, suggesting that gait dynamics provide robust social signals beyond static morphology. These findings highlight the adaptive significance of human locomotion in competitive social interactions. Future research should investigate the potential for a trade-off between static and dynamic cues to dominance, particularly in situations where static cues might be obscured.

## Introduction

Male intrasexual conflict has long presented an evolutionary selective pressure^1,2^. Aggressive interactions can result in injury and death, as is apparent from many contemporary accounts, alongside much evidence of trauma in human skeletal remains across millennia^3^. Such strong selective forces are thought to have shaped men’s phenotypic traits^4^. Prior to 2010, a substantial body of research focused on subjective perceptions of male-typical traits in the context of female mate choice (e.g. using female judgments of male physical attractiveness; review in^5^. More recently, attention has shifted towards male-typical traits interpreted through the lens of men’s intrasexual competition^4,6,7^. Men’s faces, voices and bodies all show quite distinct sexual differentiation^8–11^ and many of these traits are thought, at least to some extent, to either provide a defence against violence^12^ or help inflict it^13^. For example, it has been proposed that men exhibit thicker cranial bones and more robust mandibles^6^ as a possible means to mitigate fracture risk during physical conflict. Furthermore, the sudden stretching of active jaw muscles, for example, following a punch^14,15^, may stabilise the jaw^16–18^, absorb energy (reviewed by^19^, and reduce bone strain and concussion risk by stiffening the head-body connection and reducing brain acceleration upon impact^20–22^. Moreover, increased upper-body muscle mass has been linked to the capacity to inflict greater damage^23,24^. Indeed, both empirical data and mathematical modelling of men’s sexually dimorphic traits support the notion of intrasexual competition being the key selective force for their development^4,25,26^.

Aside from attributes that directly facilitate physical conflict, a fundamental component in successfully navigating the dangers of violence are the neurocognitive mechanisms that assess the risk that potential competitors represent^27^. Deciding whether to engage in a violent altercation should not be a trivial decision, as even the victor can sustain potentially life-threatening injuries. Further, as a deeply social species, we should anticipate a human ability to infer an individual’s formidability (i.e., fighting ability) that is not necessarily restricted to the most relevant actors (i.e., the adult male fighters) but extends to all members of the group. Indeed, even children show an understanding of conflict dynamics^28^. When assessing the visual cues to formidability from the faces and bodies of men, Sell and colleagues^24^ found that both men and women from culturally diverse samples made similar judgments when identifying physically dominant men from photographs of their faces and bodies. Of particular relevance was the degree to which men were judged to “have the physical ability to win unarmed fights, with those who have higher physical dominance winning more fights”^29^. More generally, this is linked to a large body of research that finds body size is a key determinant of knockout power (a proxy for force output) and resource holding potential across species. For example, Caton et al.,^30^ found that observers’ judgements about a fighter’s success tracked a fighter’s knockout power, and their body size mediated this relationship. It is thought that the visual cues that facilitate these judgments provide information that tracks the upper-body strength of the individual and, thus, their formidability^4,24^. Similar findings for the encoding of formidability in facial features have since broadly followed the same results^30–33^.

In the foregoing literature, participants have usually been asked to judge formidability from static stimuli. This begs the question of what role dynamic visual information might play in such judgments. Biomechanical changes in joint angles with time, leading to biological motion, may present a vibrant source of visual information for inferring differences in individual formidability. Importantly, one of the core assumptions of biological motion perception, particularly concerning perceptions of walking gait (upon which most of the human literature is based), is that it falls under unconscious control and is thus ‘honestly’ represented^34^. Much of the animal literature on courtship displays also assumes this principle, with females from multiple species using males’ performance to make mating decisions^35–37^. Indeed, in the domains of social and visual perception, abundant literature exists exploring our intuitive understanding of social agency and our ability to infer a multitude of higher-order characteristics via biological motion^38^. Much of this work utilises minimalist representations of human bodies through point-light projections, where a visual representation of a body is created by strategically placing reflective dots on central and distal body parts, such as on shoulders, elbows, and wrists. Even though point-light projections constitute impoverished stimuli, observers can deduce the target person’s gender^39^, personality traits^40,41^, affective state^42,43^, and intentions^44^. Indeed, point-light displays that are spatially scrambled still seem to contain motion patterns that act as a ‘life detector’ of sorts^45^, triggering specialised neurocircuitry in areas such as the superior temporal sulcus (STS)^46,47^. Even congenitally blind people who have their sight restored are spontaneously and rapidly able to identify human locomotive patterns^48^ and this apparently innate ability to recognise biological motion can be detected in newborns^49^.

Recent innovations in measuring and recording human biomechanics have greatly enhanced our ability to conduct such studies. Representing human biomechanics independently of other variables is challenging because the representation must eliminate possible confounds encountered in natural settings that may influence perception, such as hair, clothing, facial information, build, height, and social context. Historically, the point-light technique has played a fundamental role in the initial exploration and advancement of several research domains, but it has limitations. Firstly, this technique restricts observations to two-dimensional representations. While human binocular vision can use disparity cues to generate the perception of depth through stereopsis, standard two-dimensional point light displays do not contain disparity information, and depth must be inferred from alternative cues such as occlusion (i.e. when one body part occludes the point light placed on another body part^50^). Furthermore, in traditional point-light displays, the perception of bodily proportions exactly matches those of the subjects because the displays are direct 2D projections of the locations of the markers attached to bodily landmarks. This variability in bodily proportions provides observers with additional cues about a person’s size, which when viewed back-to-back with other point-light actors, allows for structural comparisons to be made rather than purely biomechanical ones. To solve this problem, modern methods implement algorithms which standardize features such as the distances between joints thereby removing such confounds, leaving only joint angle information^51^. In addition, current 3D motion capture techniques allow for the creation of standardised avatars with controlled body size, proportions, and appearance, thereby allowing an exclusive focus on biological motion^52,53^.

Here, we employ modern 3D motion capture techniques to ascertain the salient biomechanical characteristics that drive perceptions of men’s physical dominance. To do this, we recorded the motion of male participants walking towards a virtual viewer. We chose to focus on a steady walking gait because we wanted physical dominance to be judged from movement patterns frequently encountered in daily life rather than those reflecting specific rituals or social actions. Furthermore, extracting specific attributes (e.g., sex, mood) from biological motion displays of gait has been found to be quicker and more accurate than from more complex motion sequences^54^. The motion recordings were individually rated for dominance by a separate and larger set of both male and female participants. We then asked a panel of ten judges to identify what patterns of movement discriminated the five video sequences rated the highest and the five lowest for dominance. There was unanimous agreement that two features, swagger and sway were responsible. Sway is the degree to which men’s torsos oscillated to the left and the right, in the coronal plane, with each step as they walked. Swagger comprised consistent shoulder abduction from the chest together with elbow flexion, and internal rotation of the shoulder. Therefore, we carried out a biomechanical analysis of each video sequence to quantify the extent of swagger and sway exhibited by each walker so that we could model the relationship between physical dominance and these biomechanical markers.

Critically, we also wanted to control for the possibility of identifying a set of false positive associations between dominance ratings and the biomechanical features marking swagger and sway. Logically, in a laboratory situation, where participants are asked to judge a range of stimuli, it is possible that they might use a rating strategy and/or adapt their response scale to the specific task at hand; developing, on the fly, a heuristic, which allows them to discriminate between the stimuli in a given set, just for the sake of completing the task they have been given. For example, a participant might focus on the movement of a particular body feature, which happens to show a great deal of variation in that particular set of images and which can thus be used to efficiently differentiate between the images in their ratings. However, this may not be the strategy/feature they might otherwise use in actual judgements of formidability in real-world situations. To control for this possibility, we also obtained hand grip strength measures, as well as anthropometric data, from the participants whose 3D walking motion was captured. The rationale is that previous studies^24,55^ have shown that such information is strongly correlated with judgments of formidability and self-reports of real-world fighting. Therefore, to exclude a false positive result, we should expect that cues such as grip strength and height should correlate not only with our participants’ estimates of the walkers’ dominance but also with the biomechanical markers (swagger and sway) that associate with dominance. In a similar vein, we wanted to know if trait aggression measures^56^ reported by the walker, at the time of their 3D motion capture, were also associated both with the dominance ratings as well as with biomechanical markers. In short, by including these covariates, we could exclude the possibility of identifying arbitrary, non-meaningful associations between dominance ratings and biomechanical markers.

## Results

### PCA of anthropometric measures

We wanted to model the relationship between the dominance ratings of the walkers, the biomechanical measures that capture sway and swagger, while taking account of the potential influence of the following covariates: age and BMI of the walkers, their trait aggression scores, hand strength, and other anthropometric measurements (biceps, shoulder, chest, and waist circumferences). Table 1 shows extensive and substantial co-linearity between these covariates. Therefore, to avoid introducing variance inflation in the statistical modelling, we used PROC FACTOR in SASv9.4 (SAS Institute, North Carolina, US) to carry out a principal components analysis with varimax rotation to identify the significant latent variable(s) amongst the covariates^8,57^. The factor scores from these latent variable(s) were used in the statistical models. In line with previous guidelines^58^ the Kaiser–Meyer–Olkin (KMO) measure of sampling adequacy (which indicates the degree of diffusion in the pattern of correlations) was 0.88, suggesting an acceptable sample. One factor had eigenvalues greater than Kaiser’s criterion of 1 (i.e., 5.25), which explained 75.0% of the variance. The scree plot showed an inflexion (i.e., Cattel’s criterion), which also justified retaining just one factor. The residuals were all small, and the overall root mean square off-diagonal residual was 0.04, indicating that the factor structure explained most of the correlations. The factor loadings for the single principal component (PC1) are shown in the last column of Table 1.

**Table 1:** Shows the pearson correlations between the non-biomechanical variables from the walkers.

	BMI	Hand s	Biceps	Shldr c	Chest c	Waist c	PC1 loadings
BMI	–						0.98
Hand s	0.66***	–					0.68
Biceps	0.89***	0.64***	–				0.92
Shldr c	0.77***	0.59***	0.83***	–			0.84
Chest c	0.79***	0.49**	0.75***	0.74***	–		0.82
Waist c	0.91***	0.62***	0.81***	0.71***	0.77***	–	0.92
Hip c	0.76***	0.49**	0.60***	0.57***	0.60***	0.72***	0.73

s , strength; Shldr = shoulder; c, circumference; * *p* < .05; ** *p* < .01; *** *p* < .001.

We used the factor scores from PC1, henceforth referred to as ANTH, for the linear mixed effects modelling described below. Higher scores on ANTH are related to larger body size (indexed by BMI, biceps, shoulder, chest, and waist circumferences) and greater strength (indexed by hand grip score).

### Pearson correlations between dominance, biomechanical measures, ANTH, and BPAQ

In addition, we calculated the Pearson correlations between the dominance ratings, the four biomechanical measures taken from the walkers, ANTH and the BPAQ total scores (see Table 2). We found significant correlations between the dominance ratings and three of the four biomechanical measures (i.e., sway, shoulder abduction and internal rotation) and ANTH. Importantly, we also confirmed that ANTH was significantly correlated with these three biomechanical measures, and that BPAQ total was significantly correlated with the sway measure. These inter-correlations therefore suggest that the associations between dominance ratings and the biomechanical measures extend beyond mere laboratory artefact, and are indeed plausibly related to formidability, because the biomechanical measures and dominance are also inter-correlated with ANTH and BPAQ total.

**Table 2:** Shows the pearson correlations between dominance scores, Biomechanical variables from the walkers, ANTH and BPAQ.

	Dominance	Sway	Shoulder ABD	Elbow flexion	Shoulder IR	ANTH
Sway	0.51 **	–				
Shoulder ABD	0.67 **	0.39 **	–			
Elbow flexion	0.17	0.13	0.25	–		
Shoulder IR	0.42 *	0.066	0.63 ***	−0.15	–	
ANTH	0.63 ***	0.30 *	0.63 ***	0.034	0.44 **	–
BPAQ total	−0.20	−0.34 *	−0.036	0.045	0.17	−0.088

ABD adbuction; IR internal rotation; *p < .05; ** *p* < .01; *** *p* < .001.

### Linear mixed effects modelling

We used PROC MIXED in SAS (v9.4) to run a linear mixed effects model to predict dominance ratings from: walker sway, shoulder abduction, shoulder internal rotation, elbow flexion, age, ANTH, and BPAQ total as well as observer sex and observer age. We tested adding random effects at the intercept level for both participants as well as items, and we also tested for random slopes at the participant level for walker sway, shoulder abduction, shoulder internal rotation, and elbow flexion. We used the Satterthwaite method to estimate degrees of freedom. Rater sex was dummy coded with male raters as the control. Explanatory variables were retained in the model if they showed significant Type III tests of fixed effects, and contributed to a significant reduction in −2 log likelihood.

For random effects, the model showed significant variance in intercepts for participants, Var(u_0j_) = 0.28, Z = 5.57, *p* < .0001, and items Var(u_0j_) = 0.23, Z = 4.50, *p* < .0001. In addition, the slopes varied across participants for ANTH, Var(u_1j_) = 0.047, Z = 4.45, *p* < .0001, and shoulder abduction, Var(u_1j_) = 0.00060, Z = 2.04, *p* = .02. Finally, the participant slopes for ANTH and participant intercepts negatively and significantly covaried, Cov (u_0j_, u_1j_) = −0.057, Z = −3.14, *p* = .022. We found statistically significant fixed effects of sway (F1, 49 = 2.35, *p* = .02, β = 0.037, SE = 0.016), shoulder abduction (F1, 45.6 = 45.6, *p* = .006, β = 0.077, SE = 0.0064), and ANTH (F1, 49 = 2.68, *p* = .009, β = 0.25, SE = 0.092). This suggests that greater sway, shoulder abduction and strength/body-size independently contribute to greater dominance scores. In addition, we also found a significant influence of rater age, whereby the older a rater was, the higher was the dominance rating they assigned (F1, 125 = 2.03, *p* = .04, β = 0.013, SE = 0.0064). There were no statistically significant interaction terms in the model. Moreover, there were no independent significant contributions to the model from shoulder internal rotation, elbow flexion, BPAQ total, or rater sex.

In the present study, f^2^ = 0.071 for the overall model indicating sway, shoulder abduction, ANTH, and rater age explain 6.6% of the variance in dominance scores relative to the unexplained variance in dominance scores. Guidelines for interpretation of f^2^ indicate that 0.02 is a small effect, 0.15 is a medium effect, and 0.35 is a large effect^59,60^ indicating that the present effect is small to medium.

**Fig. 1:**
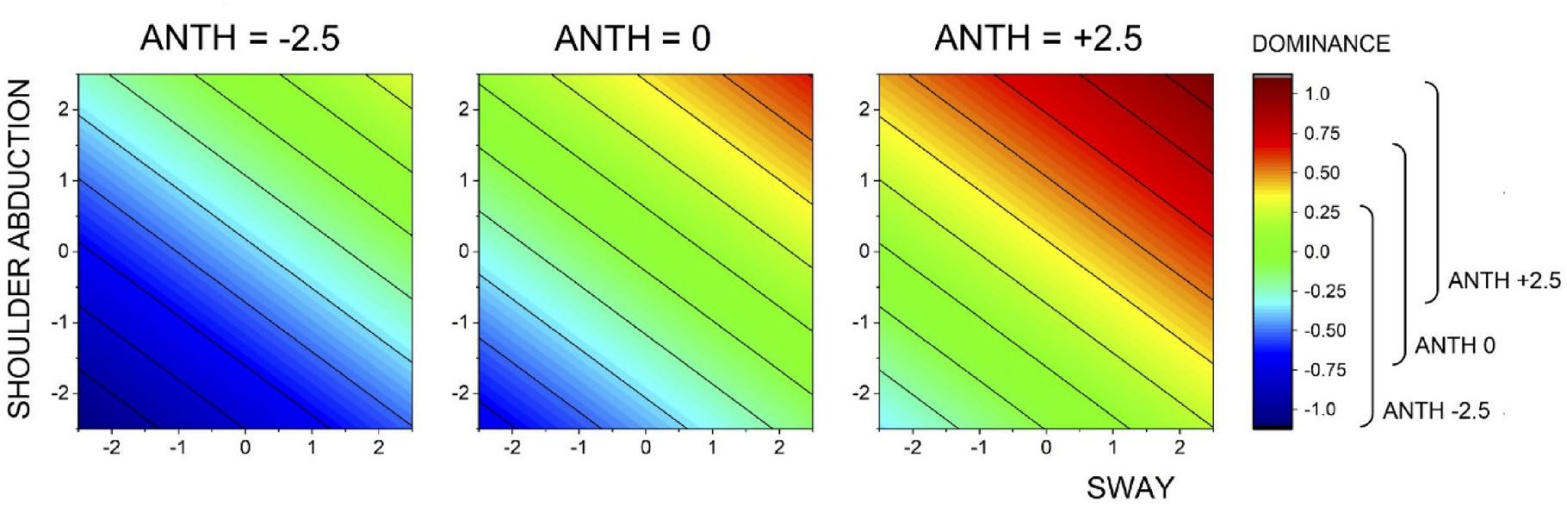
Heat maps at 3 levels of ANTH, each showing how dominance scores increase linearly as a combined function of shoulder abduction and sway. The brackets on the dominance colour scale illustrate the increasing range of dominance covered for ANTH = −2.5 SD, 0SD, and + 2.5 SD, respectively.

Figure 1 is a graphical illustration of the model outcome. It shows three heatmaps, one calculated at three levels of ANTH: −2.5 SD, 0 SD, and + 2.5 SD. In each case, model-predicted dominance scores are plotted as a function of shoulder abduction (y-axis) and sway (x-axis). Figure 1 shows the additive effects of increasing shoulder abduction, sway and ANTH on increasing dominance scores. Moreover, the iso-contours in the 3 heatmaps make it clear that different combinations of these three variables can predict equivalent dominance ratings.

## Discussion

Our study employed modern 3D motion capture techniques to ascertain the salient biomechanical characteristics of men’s gait that are associated with perceptions of their physical dominance and to validate these perceptions against the target men’s size, strength, and trait aggression. Accordingly, we found substantial correlations between: physical dominance (indexing formidability), three biomechanical parameters (sway, shoulder abduction and internal rotation), a principal component that encoded physical size and strength (i.e., ANTH), and men’s trait aggression. In our linear mixed effect model predicting dominance scores, where explanatory variables are effectively competing against each other, the final model retained independent contributions from two biomechanical parameters: sway and shoulder abduction, together with physical size and strength, and a contribution from raters’ age. Trait aggression no longer made an independent contribution to the final model presumably because the variance it shared with dominance ratings was also shared, and subsumed by, the biomechanical feature sway.

To estimate physical dominance, evidence supports the hypothesis of an evolved cognitive architecture that integrates information from a variety of visual and auditory sources such as faces, bodies, and voices^4,24,61,62^. However, estimating physical dominance from visually static cues alone will exclude dynamic information that may account for additional variance in fighting ability, such as motor dexterity and cardiovascular fitness^2^. Moreover, impediments such as poor lighting or bulky clothing may further mask our ability to parse static visual scenes which are replete with real word objects that may also partially occlude each other. Fortunately, substantial social perception literature (see^38^ for an overview) suggests that biological motion, a dynamic cue, can provide a rich source of information regarding animacy, agency and intention, all of which can be captured at a glance, even from impoverished stimuli such as scrambled point-light displays which are devoid of explicit physical form^63^. There is also evidence to suggest that specialised neural circuitry in the superior temporal sulcus (STS)^46,64^ participates in extracting high-order features from the normal walking gait. Consistent with this, behavioural research by^65^ showed that both men and women can accurately assess male strength through gait, and women from different cultures tend to find stronger men’s gaits more attractive. Similarly^52^, showed that humans can infer physical strength from other biological motion cues like dancing. However, it is important to note that these phenomena may not be universally applicable^66^ suggesting the presence of potential social factors intertwined with the detection of physical strength from gait. Nevertheless, taken together, research suggests that biological motion would be an exemplary candidate for detecting socially consequential characteristics such as physical dominance.

Our findings indicate that biomechanical features extracted from men’s gait, as well as their body size and strength, are all positively correlated with the assessment of their physical dominance, when each is considered in isolation. Perhaps more interestingly, the linear mixed effects modelling, which had these different features competing against each other, settled on an additive model of physical dominance, as illustrated by Fig. 1. Essentially, increases in three features: sway, shoulder abduction, and body size / strength sum together to predict a given level of physical dominance. Critically, this four-dimensional parameter space means that the same level of physical dominance can be achieved in a variety of different ways. For example, two men of the same body size and strength could represent the same physical dominance either by exhibiting marked shoulder abduction with modest sway, or modest shoulder abduction with marked sway. Intriguingly, the data also permit that, by employing marked shoulder abduction and sway, smaller weaker men could be perceived as having equivalent physical dominance to a substantially larger and stronger man who exhibits negligible shoulder abduction and sway; akin to a phrenetic David up against a placid Goliath.

Reassuringly, our findings are consistent with prior literature. For example, from a biomechanical point of view, the combination of sway and shoulder abduction would create the stereotypical walk of the Western hero, as described by^39^. Similarly, the same features to a large extent define the very ‘male’ walk described by^67^. Satchell and colleagues found that both increased rotational movement, with respect to a fixed horizontal plane, between the upper and lower body as well as faster gait speed in men were positively correlated with aggression^41^. We note that it is plausible that Satchell et al. may have captured components of the same sway metric that we describe in the current study.

From our results, we suggest that the spectrum of maleness identified in^39^ be scaled by a man’s physical dominance characteristics (i.e., his size and strength). The makeup of these variables contains both a structural/postural component, in the case of swagger, and a dynamic component, in the case of sway. This combination marries well with much of the biological motion perception literature that suggests that we can more easily resolve the global human form by combining structural/postural information with the figure’s temporal properties^68,69^. The ability of the visual system to achieve a fast, highly robust depiction of a human in locomotion is thought to combine bottom-up, low-level vision processing with predictive “visual routines” in a top-down manner^69,70^. In the case of the former, it is thought that the low-level processing can operate almost passively, with very little sensitivity to attention, thus providing a compelling format to establish potential danger.

From an evolutionary perspective, it should benefit individuals to be able to detect those who have the capacity to inflict serious harm quickly and accurately. In terms of processing speed, static cues such as body size and muscle mass (if available) should be estimable more quickly than dynamic cues such as sway because the latter requires information to be integrated over time. Therefore, for situations that are time-critical, is there redundancy in the three cues we have identified? Size and strength (encoded as ANTH here), as well as shoulder abduction, are arguably relatively static cues which can be estimated with a brief glimpse. However, sway is a dynamic cue that could potentially take seconds to estimate. So, is sway needed?

To address this, first we should re-emphasize that, in the present study, the only information available to the raters in this study concerned the posture of the avatar, together with dynamic biological motion cues. No information about body size, strength, or trait aggression was directly available to raters. Yet these latter features played a statistically significant part in accounting for the dominance ratings we obtained. This suggests that, at least in part, such information can be embedded in complex gait patterns (see e.g^65^),and consequently, the argument could be made that motion cues alone may suffice.

However, with regard to static size/shape cues that are explicitly available, various species engage in behaviours to ascertain the physical dominance of a competitor before engaging in potentially lethal combat, often termed ritualised aggression^13^. For example, red deer will size their opponents up using increasingly direct means before engaging in a more lethal confrontation^71^. In humans, the upper body muscles in the chest, shoulders and arms are crucial for dealing damage in unarmed combat, and research has shown that visual inspection of the torso is integral to perceptions of formidability^24^. Consequently, a fight can be avoided if there is sufficient size disparity between participants^72,73^ and apparent size differences can also be exaggerated. For example, pufferfish inflate their bodies dramatically to deter potential predators^74^. Similarly, our data showed that consistent shoulder abduction led to higher ratings of dominance from observers. This postural component (seen in Fig. 4) may have acted to exaggerate the apparent width of the upper body.

If apparent size is a compelling and potentially sufficient cue to formidability, what further advantage might sway bring? We suggest that sway may provide cues to formidability in situations where static cues may be partially masked. For example, other people in a crowd, vegetation in the immediate vicinity, clothing, ambiguous lighting (e.g. when someone approaches with the sun behind them) may all partially or even completely mask an individual’s shape and posture, rendering static cues less effective at transmitting formidability. Therefore, under such circumstances, we speculate that sway might transmit a slower, but nevertheless robust code, particularly in light of the literature showing that biological motion is robust to random noise^75^. Clearly, future research could be aimed at testing potential trade-offs between explicit size/strength cues, postural cues, and biological motion cues.

Thus far, we have considered the relationships between biomechanical variables, physical size and strength in relation to the perceived physical dominance of the walker, from an honest signaling perspective. This assumes that cues projected by the walker are a well-calibrated reflection of the damage they could inflict in the event of conflict^76^. However, one should consider the possibility that some individuals might engage in dishonest signalling whereby the cues projected by the walker are a misleading overrepresentation of their actual capacity for inflicting harm. The role of dishonesty remains a contentious topic in the animal communication literature^76,77^ with theoretical models indicating that the most evolutionarily stable strategy is one where, on average, signals or cues are honest^78^. Instances where dishonesty is more common should favour those who ignore such signals, leading to a breakdown in communication between the signaller and the receiver. Nonetheless, empirical evidence suggests that dishonesty is more prevalent than one might expect, particularly in signals of physical prowess intended to deter fighting. Dishonesty would play a stronger role where the costs associated with engaging in conflict are extremely high, and assessing physical dominance is difficult without ritualised aggression (e.g., in arthropods)^76^. As mentioned above, our model permits weaker men to employ exaggerated sway and shoulder abduction as a means to appear more dominant. This could be a mechanism somewhat under conscious control to avoid the onset of altercations in a form of ‘intimidation dynamics’. In theory, this should only marginally increase perceptions of dominance to mitigate the costs associated with a genuine altercation with a significantly stronger man, maintain the reliability of the cue, and because kinematics are hard to fake reliably^34,35^.

Another consideration is where the intention is not to avoid conflict, but to actively engage in it. For example, for an assassin to successfully strike their target, they might need to engage in close-quarters combat. However, on their approach to the target, they must remain undetected. Interestingly, we found that BPAQ aggression scores^56^ which arguably reflect aggressive intent, were inversely correlated with both dominance scores and, particularly, the magnitude of sway. In a similar vein, if one’s internal state shifts towards engaging in a violent encounter, their kinematics will need to change to ensure a stable centre of mass and physical readiness. This would clearly be useful during violent encounters, aiding both offence, in the form of providing a stable position with which to strike, and defence, to avoid being knocked off balance. Under these circumstances, the particular movement patterns we have identified, such as sway, may mitigate against this. We recognise that this remains speculative at present, yet it presents an intriguing direction for future research.

Finally, as a cautionary note, we emphasise that inferences regarding dynamic cues in the current study apply only to motion-captured data from normal walking. Other research has considered other kinds of dynamic and postural cues that are relevant in a variety of contexts. For example, the degree to which an individual’s posture is perceived as erect is strongly associated with the perception of physical dominance^79^. Further, following victory in Olympic medal matches, winners often display reflexive whole-body displays such as throwing one’s fists in the air and open arm expansion to signal triumph, which in turn signals dominance^80^.

To conclude, in this motion capture study of men walking, we have replicated previous studies that have shown that judgements of these men’s physical dominance (formidability) are associated with their strength, size, and trait aggression. We can add two new components that contribute to these complex judgements: a postural cue captured by shoulder abduction and a dynamic cue captured by the degree of mediolateral sway of the thorax.

## Methods

All methods were carried out in accordance with relevant guidelines and regulations. All experimental protocols were approved by the Department of Psychology Ethics Committee at Northumbria University, on 16/06/2014. The Reference code for the ethics proposal was:

SUB05_KM. Informed consent was obtained from all participants and/or their legal guardian(s).

### Motion capture: walker participants

Sixty men aged 18–41, 6 self-reporting non-White ethnicities, were recruited to the study to have their walking motion captured. None reported any illness or injury that could interfere with their movements. Due to various data capture and processing issues (e.g., excessive marker occlusion), a final sample of 52 men was used.

We collected anthropometric and behavioural data to capture the physical dominance characteristics of each man. Flexed biceps circumference, shoulder width, chest width, waist width and hip width^4^ were assessed using a Seca measuring tape. Using a Jamar Handgrip Strength Dynamometer, we also collected maximal force handgrip strength (as a proxy for body strength^81^. This was performed twice per hand, and an average was taken. Each man also completed the Buss Perry Aggression Questionnaire (BPAQ)^56^. This psychometric task comprises twenty-nine items scored on a five-point Likert scale from 1 (extremely uncharacteristic) to 5 (extremely characteristic). The test comprises four subscales: (a) Physical aggression (behavioural), i.e., the tendency to engage in physical acts of aggression, which is based on nine items, e.g. “If I have to resort to violence to protect my rights, I will”; (b) Verbal aggression (behavioural), i.e., the propensity to engage in verbal arguments and confrontations which is based on five items, e.g. “I can’t help getting into arguments when people disagree with me”; (c) Anger (affective), i.e., the emotional aspect of aggression, including quick temper and frustration which is based on seven items, e.g., “I flare up quickly but get over it quickly”; (d) Hostility (cognitive), i.e., feelings of ill will and suspicion towards others​​​​​​ which is based on eight items, e.g. “I wonder why sometimes I feel so bitter about things”. The four sub-scales are also compiled into a total BPAQ score, which shows high reliability, with Cronbach’s alpha between 0.86 and 0.93^56,82,83^. The descriptive characteristics of the male walkers are described in Table 3.

**Table 3:** Characteristics of the 52 male walker targets.

Participant characteristics	M	SD	Range
Age (years)	22.56	4.04	18.00–41.00
Anthropometric measures			
BMI	22.95	3.77	15.16–33.02
Hand strength (kg)	43.23	7.42	30.00–62.00
Biceps circumference (cm)	33.32	4.61	23.00–48.50
Shoulder circumference (cm)	111.76	8.21	92.50–136.00
Chest circumference (cm)	94.65	10.03	59.50–118.00
Waist circumference (cm)	81.48	7.70	62.00–102.00
Hip circumference (cm)	88.92	7.98	63.00–106.50
Psychometric measure			
BPAQ physical aggression	22.37	7.85	9.00–39.00
BPAQ verbal aggression	15.58	4.36	5.00–25.00
BPAQ anger	16.54	5.74	7.00 - 29.00
BPAQ hostility	20.63	6.60	8.00–34.00
BPAQ total	75.12	19.77	34.00–111.00
Biomechanical measures			
Sway	14.63	4.87	5.35–25.53
Shoulder abduction (degs)	10.93	3.66	3.61–17.06
Shoulder internal rotation (degs)	8.40	13.30	34.12–34.28
Elbow flexion (degs)	36.75	5.42	27.90–64.81

### Motion capture: measures and procedure

For accurate kinematic analysis, anthropometric measurements of each man were made including height (mm), weight (kg), leg lengths, ankle widths, knee widths, elbow widths, and wrist widths (all mm).

A 14-camera Vicon MX optical motion capture system was used to capture all movement data (T-Series, Vicon Motion Systems, Oxford) running Nexus v1.7 software. Each camera captured frames at 200 Hz. Thirty-eight 14 mm reflective markers were attached to each participant in accordance with the Plug-In-Gait kinematic model manual (Vicon, Oxford). Participants were initially requested to perform a T-pose (standing upright with arms held horizontally to the sides of the body) for a static calibration and to ensure accurate marker placement. Following their calibration, participants were asked to walk up and down an eight-metre walkway. Participants were told this was to perform a ‘dynamic calibration’ when in reality, this was to allow them to relax into their natural gait pattern as much as possible. After around five minutes of walking up and down the walkway, six one-way recordings were made without alerting the participant. Immediately after the motion capture, participants were debriefed and provided with full information on the recordings that had been made. They were also asked if they wanted to remove their data from the dataset; none chose to do so. The resulting optical data were cleaned and processed in Vicon Nexus.

**Fig. 2:**
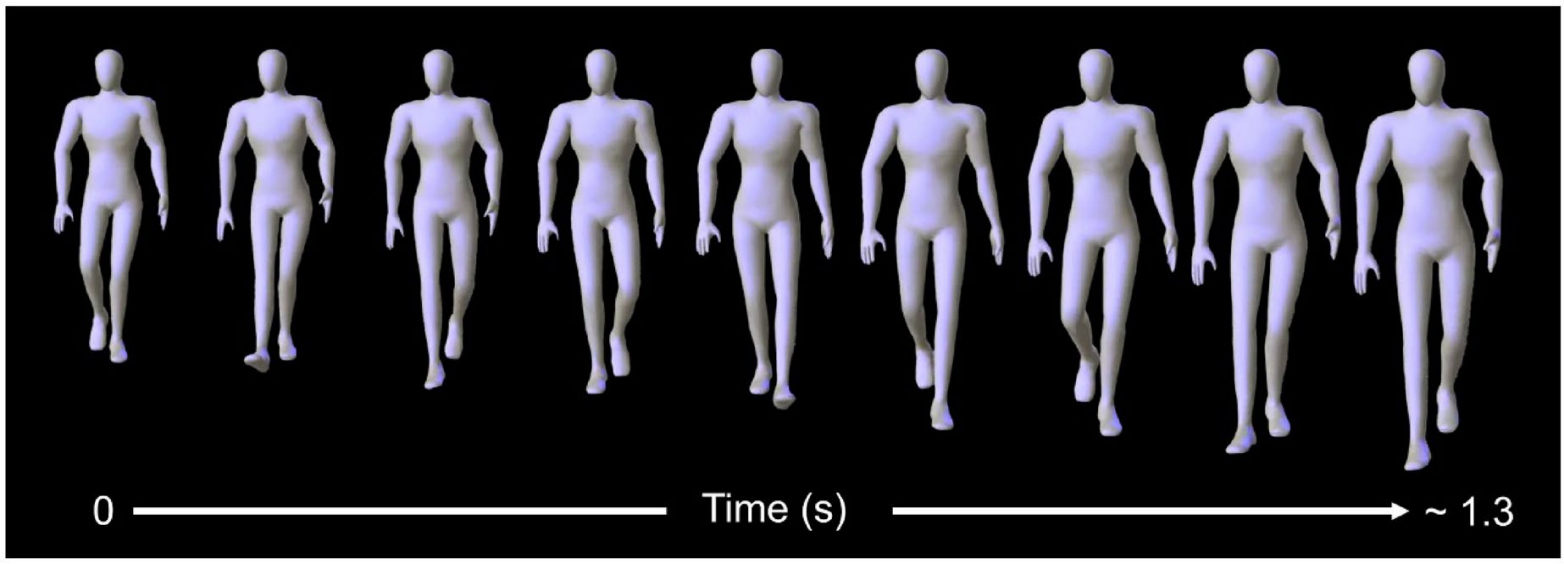
Illustration showing every fifth frame from animation sequence of standard avatar walking towards the rater.

Of the six one-way recordings, one of the latter three was selected to be rendered and shown to raters. Videos were rendered to approximately two full gait cycles (four steps) and lasted between three and four seconds. The recording was chosen based on two criteria: (a) data quality, i.e. the recording with the fewest gaps, which could not be rectified via linear interpolation. Typically, this tends to be where a marker is missing for ~ 30 consecutive frames or more. (b) lack of distinctiveness, for example, walks where the walker turns their head or adjusts their clothing would be excluded. Only the selected recording was subject to biomechanical analysis. These walks were then exported to C3D format for the creation of height- and build-standardised, featureless humanoid avatars in Autodesk MotionBuilder 2015 (Autodesk, San Rafael), as illustrated in Fig. 2.

### Motion capture: biomechanical analysis of walker motion

The key aim of this study was to ascertain the salient biomechanical characteristics that drive dominance ratings. However, with the scope of biomechanical properties being virtually limitless, we initially undertook a scoping procedure whereby ten naive participants (6 F, 4 M) from the department of psychology viewed 10 videos simultaneously on a screen, with the 5 walks rated least physically dominant (range 2.34–2.72 mean dominance rating) presented at the top of the screen, and the five walks rated most physically dominant (range 4.88–5.25 mean dominance rating) at the bottom of the screen. These 10 participants were asked to give a free-text description of what differentiated the two rows of videos and to be as specific as they could when referring to the movements. Their feedback was used to hone in on two salient features that observers all agreed discriminated clearly between the two sets of video clips: sway and swagger.

#### Sway

**Fig. 3:**
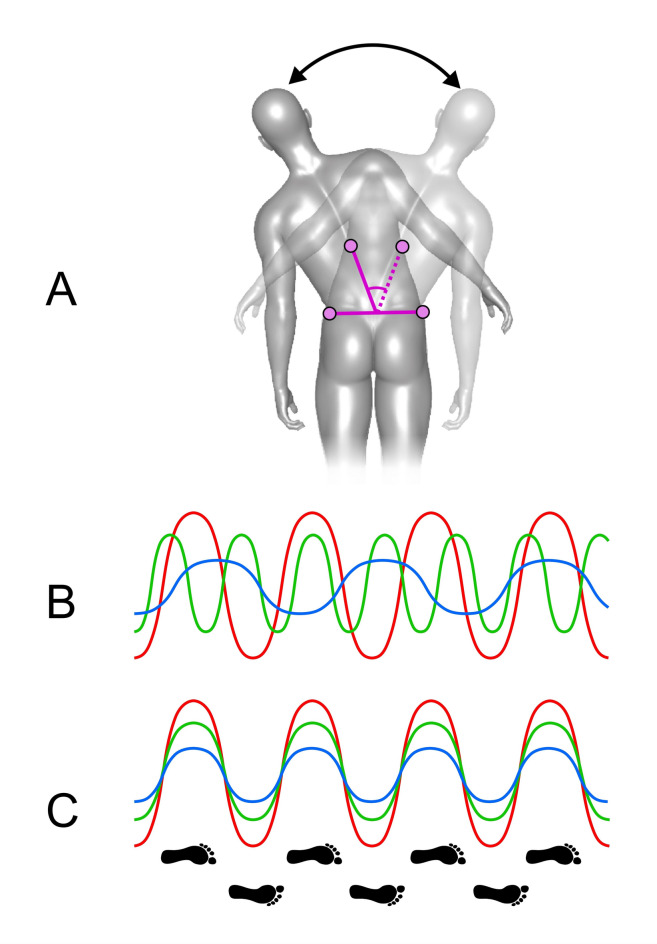
(**A**) Illustration of sway. T10 and T10’ represent the 10th thoracic vertebral marker at the two extremes of sway. LPSI and RPSI represent the left and right posterior superior iliac spine markers. (**B**) illustrates how raw values of sway varied in both amplitude and frequency as a function of time. (**C**) illustrates how the co-registration to a common step length isolated the variation in amplitude only, by resampling the time series.

The first feature we describe henceforth as sway. It is the degree to which men’s torsos oscillated to the left and the right, in the coronal plane, with each step as they walked. The feature is illustrated in a somewhat exaggerated fashion in Fig. 3A. To capture this movement, we computed the difference in horizontal excursions in the coronal plane (measured in millimetres) between the 10th thoracic vertebral marker and the mean distance between the left and right posterior superior iliac spine markers, as a function of time. Note that we normalised the distance between these two points across walkers to take account of differences in their heights. Sketches of raw data are illustrated in Fig. 3B. This shows that sway varied in amplitude and frequency as a function of time, owing to different men having different pace lengths and walking speeds. To isolate the variation in amplitude and our desired signal of interest, we therefore resampled the time series in such a way as to allow all walker’s steps to be co-registered to a common distance, as illustrated in Fig. 3C. From the co-registered data, we then calculated the average, maximum range, and standard deviation of the sway metric per walker (measured in millimetres).

#### Swagger

**Fig. 4:**
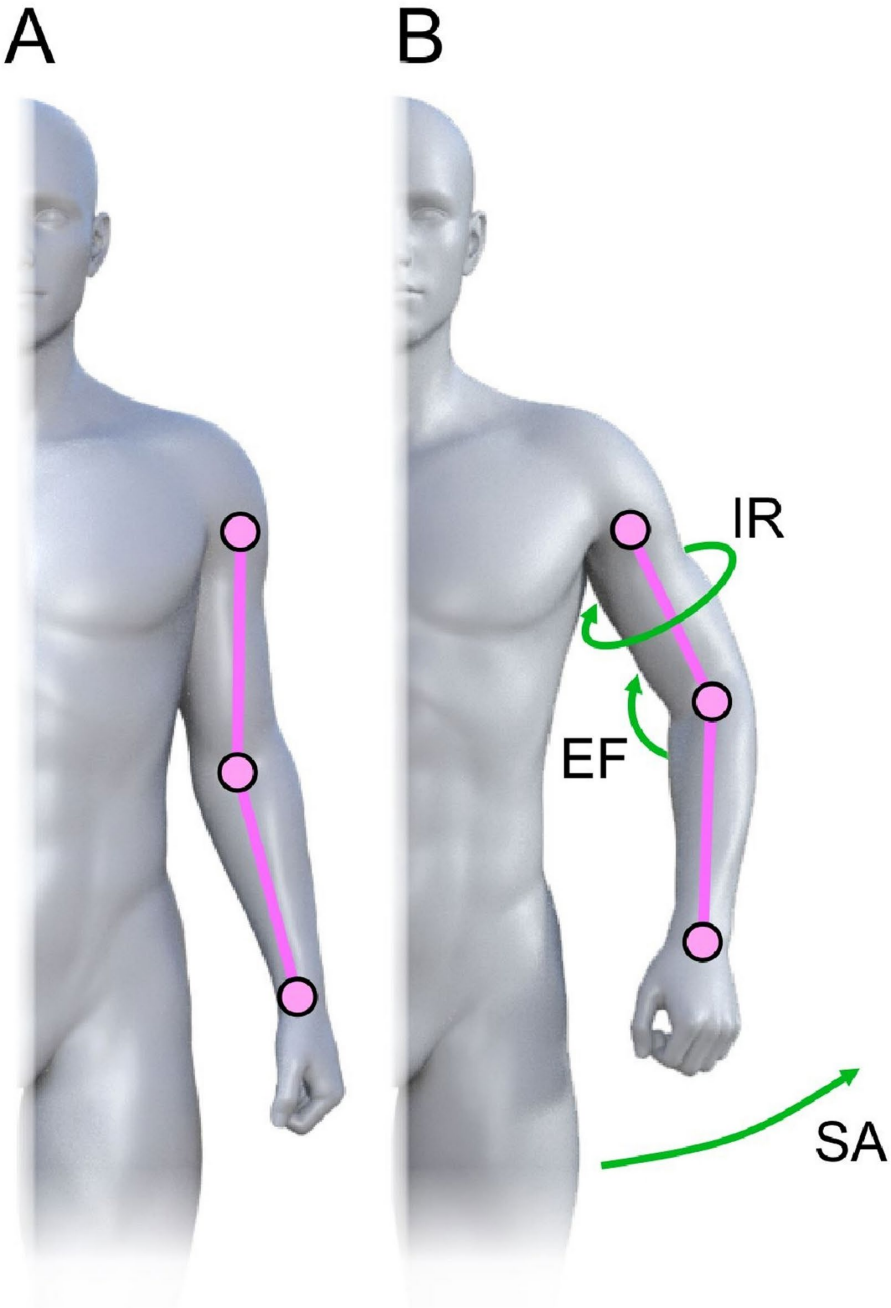
(**A**) shows the left arm in a neutral position. (**B**) shows the left arm in a swagger posture which comprises three joint angle changes including: (a) abduction of the shoulder (SA) away from the torso, (b) internal rotation (IR) of the shoulder, and (c) flexion of the elbow (EF).

The feature we describe henceforth as swagger is illustrated by the postural differences in Fig. 4. For the Vicon system, output angles for all joints are calculated from the YXZ Cardan angles (in degrees) derived by comparing the relative orientations of the segments proximal (parent) and distal (child) to the joint^84^. At one extreme, men simply walked with their arms straight, swinging their arms back and forth, with the elbows close to the abdomen (Fig. 4A). In comparison, individuals who walked with a marked swagger tended to have the shoulder consistently abducted from the chest, the elbows somewhat flexed, and the shoulder internally rotated. This posture created the appearance of a walker having an inflated upper body. Therefore, as indices of swagger, we extracted the mean, maximum range and standard deviation for three joint angles (in degrees) separately for each walker: shoulder abduction (SA), upper arm internal rotation (IR), and elbow flexion (EF), as illustrated in Fig. 4B.

### Dominance ratings: participants and procedure

One hundred and thirty-seven raters (65 F, 70 M, 2Other) aged 18–56 (M = 37, SD = 8.3) took part in the rating procedure online via Qualtrics. Raters were recruited on Prolific (prolific.ac) and were paid £3.13 for their time. Walking videos were hosted via YouTube and embedded into the Qualtrics question using an iFrame in a 500 by 375-pixel window to aid compatibility between devices and browsers. Videos were displayed in the centre of the screen with the rating questions centred below.

Raters viewed all 52 walks in a serial randomised order, rating each in terms of “How physically dominant is this man?” on a 1–7 Likert scale (1 = not physically dominant at all, 7 = very physically dominant). Raters were given a definition of physical dominance, which was described as the regularity of winning or losing a fight, with those who have higher physical dominance winning more fights. Mean dominance ratings ranged from M = 3.72, SD = 1.61, Range = 1–7, for female raters, and M = 3.56, SD = 1.55, Range = 1–7, for male raters. The average time taken for this task was around 34 min. Ratings exhibited excellent reliability (Cronbach’s α = 0.97).

## Data Availability

The data and materials for this study can be found on the OSF. A pre-registration was also created on the OSF and can be found at https://doi.org/10.17605/OSF.IO/REH4Y.
